# Treatment of Animal Tungiasis: What’s New?

**DOI:** 10.3390/tropicalmed8030142

**Published:** 2023-02-27

**Authors:** Katharine Costa dos Santos, Paula Elisa Brandão Guedes, Jamille Bispo de Carvalho Teixeira, Tatiani Vitor Harvey, Renata Santiago Alberto Carlos

**Affiliations:** 1 Departamento de Ciências Agrárias e Ambientais, Universidade Estadual de Santa Cruz (UESC), Ilhéus 45662-900, Brazil; 2 Department of Veterinary Integrative Biosciences, Texas A&M University, College Station, TX 77843, USA

**Keywords:** animal tungiasis, control, drug, zoonosis

## Abstract

In tropical and subtropical countries, particularly in disadvantaged communities, tungiasis is a severe public health problem, which is often neglected by the authorities. The sand fleas *Tunga penetrans*, predominant in endemic areas, and *Tunga trimamillata*, whose cases in humans are less frequent, are the cause of this zoonosis. Domestic animals are potential reservoirs and disseminators of tungiasis, so controlling their infection would significantly advance the prevention of human cases. This literature review compiles the most recent studies and innovations in treating animal tungiasis. Studies of approaches to the treatment of animal tungiasis, as well as disease control and prevention, are described. Isoxazolines are highlighted as promising drugs to treat animal tungiasis, with high efficacy and pharmacological protection. The positive impacts of this discovery on public health are also discussed, since dogs are an essential risk factor for human tungiasis.

## 1. Introduction

Frequent infections caused by fleas belonging to the genus *Tunga* spp. directly affect the lives of communities in endemic areas in Latin America and sub-Saharan Africa [1,2]. Tungiasis mainly poses problems in favorable climatic conditions, such as in tropical regions, especially in dry and sandy soils in poor communities, such as on the outskirts of cities, in slums, fishing villages, and rural and indigenous communities [3]. Recently, tungiasis was included on the list of neglected tropical diseases by the World Health Organization (WHO) and the Pan American Health Organization, classified in the group of scabies and other parasitic skin diseases [4], given its importance in endemic areas.

*Tunga penetrans* is the zoonotic species most often associated with tungiasis in humans and domestic and wild mammals [5,6]. Cases involving *Tunga trimamillata* are less frequent than those caused by *T. penetrans* [7]. Although, most of the time the host has a high parasite load, *T. penetrans* infections usually are self-limited [8]. Lesions caused by *T. penetrans* predispose to secondary bacterial infections [9,10], which may progress to deformity, loss of digits, self-mutilation, septicemia, and death [11,12].

Direct contact of the host with contaminated soil predisposes adult female fleas to penetrate the skin, usually in the region of the feet and hands in human cases, and the pads in affected animals. After penetration, female fleas undergo hypertrophy, forming neosomes, which mature and lay their eggs, remaining in situ until the parasite’s death, which occurs four to six weeks after penetration [13,14]. The maintenance of *T. penetrans* in the environment occurs mainly through reservoirs, including dogs, cats, pigs, cattle, and rodents, which spread the parasite eggs in the soil [8,9,10,11,12,13,14,15]. This fact contributes to human cases of tungiasis, since most of these reservoirs are domestic animals that live directly with humans, thus being a risk factor for the disease [16].

**Figure 1:**
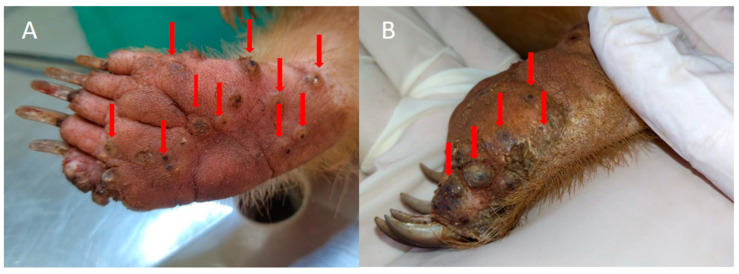
Anteater with *T. penetrans* in an endemic community for tungiasis in Brazil. (**A**,**B**) Paws of an anteater with numerous viable lesions caused by *T. penetrans.* Photos by Institute for Research and Conservation of Anteaters in Brazil.

Wild animals can also be seriously affected by tungiasis, as indicated in reports of infections in anteaters [17,18] (Figure 1), monkeys [19], and jaguars [20]. However, little is known about the importance and relationship of these wild species with human tungiasis, as well as the maintenance of the cycle of the flea *T. penetrans*, the epidemiological profile and the treatment of tungiasis in these wild species. This has given rise to the need for further studies of the disease in these species, because they can act as potential disseminators of these fleas, since most exotic species travel through large areas and are mainly prevalent in rural zones and indigenous communities that are potentially endemic areas for tungiasis [3,17,18,19,20].

**Figure 2:**
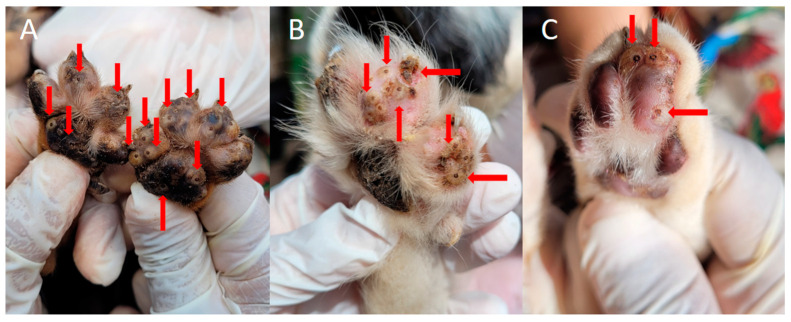
Animals infected with *T. penetrans* in an endemic community for tungiasis in Brazil. (**A**) Hind paws of a puppy with numerous viable lesions caused by *T. penetrans*. (**B**) Paw of a dog infected with several fleas, arrows demonstrating tungiasis lesions. (**C**) Cat paw with lesions compatible with viable tungiasis. Photos by Katharine Costa dos Santos.

Therefore, controlling *T. penetrans fleas* in animals can directly contribute to the prevention of human tungiasis [21], since animals favor the transmission and persistence of fleas in the environment [15,22]. In Brazil, dogs are considered one of the main carriers of fleas in the environment, while in African countries, pigs play this role [8,12]. As mentioned above, after the maturation of the eggs in the lesions present in the parasitized hosts, the eggs are released into the environment, perpetuating the life cycle of fleas [15,16,17,18,19,20,21]. Due to the close relationship of environmental sharing between these animal species and humans, people are more exposed to tungiasis in places where infected hosts are present. These factors demonstrate the importance of treating infected animals, since reducing this parasitism will consequently reduce cases in humans, since there will be less or even no dispersal of fleas, which are the causative agents of tungiasis [2,8]. This would be a good strategy for public health authorities, through policies to control fleas in potential animal reservoirs. However, such policies cannot exclude other risk factors for tungiasis, such as poor housing conditions, substandard community hygiene, and traditional techniques for treating and controlling tungiasis [2,21,23]. Thus, control must be multifactorial and involve environmental and host control [15]. Manual flea extraction and treatment with topical antibiotic therapy to prevent complications is recommended for humans [3,9,10,11,12,13,14,15,16,17,18,19,20,21,22,23,24]. However, this method does not apply in most cases of animal tungiasis, since most animals are severely parasitized [21,22,23,24,25], as seen in Figure 2.

Concerning drug treatments for animals, which is discussed in this article, this occasionally has been used in animal tungiasis, such as the topical application of the organochlorine lindane (gamma-hexachlorocyclohexane) in pigs [26,27], the topical use of trichlorphone at 0.2% (Neguvon) in infested dogs [28], a 97% oily trichlorphone solution for dogs and cats, and collars impregnated with propoxur (carbamate), and flumethrin (pyrethroid) in dogs [22]. Other topical drugs have also been tested but showed toxic and carcinogenic potential [29,30]. There are also reports of using ivermectin to treat tungiasis in dogs [31,32]. However, controlled studies proving the effectiveness of drugs to treat and protect animals against *T. penetrans* are scarce [33]. Currently, a new perspective has emerged in treating tungiasis in dogs, the use of isoxazolines (e.g., fluralaner), which have demonstrated high efficacy [34,35].

This literature review provides a comprehensive critical assessment of the literary evidence regarding the treatment and prevention of animal tungiasis, as this disease is still a global public health challenge [6,36], presenting high morbidity and contributing to serious health problems in the affected individuals [16].

## 2. Challenges in the Treatment and Control of Animal Tungiasis

Tungiasis is a tropical parasitic disease that is generally neglected by public authorities, health professionals, and the pharmaceutical industry, even though it affects millions of individuals on different continents [6]. Public policies are scarce, and there needs to be more epidemiological, geographic, and clinical data on this zoonosis, which mainly affects socioeconomically excluded people [14,37]. Demographic and behavioral characteristics of the population directly influence tungiasis morbidity in endemic regions [2]. Therefore, the association of different prophylactic measures, contemplating the control of the main risk factors for tungiasis, is an assertive alternative to control this disease [21,38].

For effective tungiasis control, it is necessary to understand the dynamics of the *T. penetrans flea* in the hosts and its development in the environment [12,13]. Flea penetration occurs on average within two days (stage I); after this period, abdominal hypertrophy begins (stage II), reaching maximum hypertrophy 2 to 3 days after complete penetration, with the formation of a white halo around the lesion 6 to 7 days after penetration (stage III). Subsequently, the lesion progresses to stage IV, which can remain for 3 to 4 weeks after penetration. At the end of the fourth week, the healing process begins (stage V), which can last until the end of the fifth or sixth week. During stages III and the beginning of stage IV, the parasite eggs are released and dispersed in the environment [14]. In the soil, the eggs hatch into larvae that feed on organic debris and develop, enabling reinfection to completing the cycle [13].

**Figure 3:**
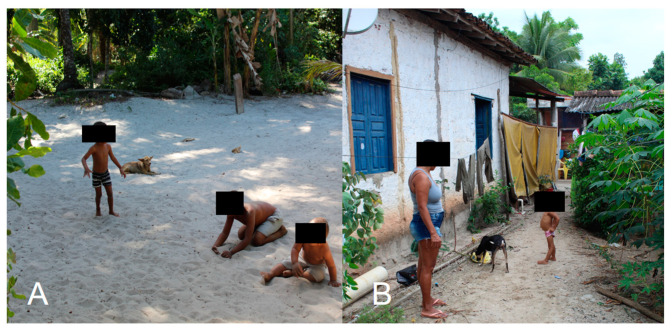
Relationship of environment, animals, and humans in a Brazilian community that is endemic for tungiasis. (**A**) Barefoot children playing on sandy soil next to a dog with tungiasis. (**B**) Barefoot child next to an infected dog. Photos by Katharine Costa dos Santos.

Sanitary and basic hygiene education is essential regarding tungiasis. For example, the habit of wearing shoes can increase the protection of the foot region against flea attacks in humans. However, in many endemic communities, this practice is uncommon [38,39], as seen in Figure 3. Moreover, the lack of paving, basic sanitation, selective garbage collection, and infrastructure in endemic areas contributes to the development of the parasite, since it adapts well to sandy soil and feeds on decomposed organic material [2,36]. Control measures must address all these issues, but the pharmacological control of fleas in reservoirs can promote a cascade effect, including control of the parasite in the environment and humans [15,16,17,18,19,20,21,22,23,24,40].

The climatic factor is also a significant challenge, since in some regions, the parasite’s presence and reproduction occur in all seasons of the year [41], unlike others where peak infection mainly occurs in the dry period [42]. Thus, in regions with no seasonality for the disease, care needs to be constant, demanding higher investment and a better control strategy by public authorities and the affected population.

**Figure 4:**
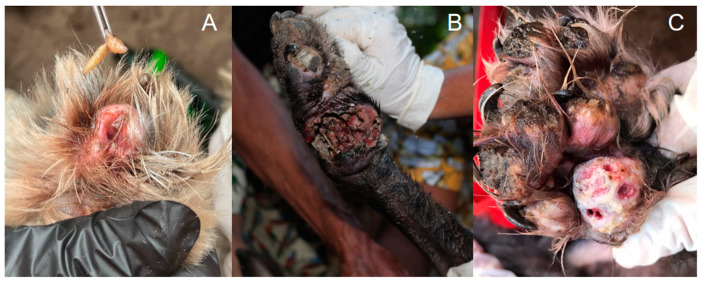
Complications of canine tungiasis. (**A**) Presence of myiasis in a lesion caused by *T. penetrans* in the paw of an infected dog. (**B**) Dog paw with tungiasis associated with secondary bacterial infection with necrotic tissue. (**C**) Dog paws with *T. penetrans* lesions showing suppuration and secondary infection. Photos by Katharine Costa dos Santos.

Another challenge in tungiasis control is that in endemic regions, it is common for most domestic animals to be semi-domiciled, with free access to the community, which enables infected animals to spread *T. penetrans eggs* throughout the environment [43]. Additionally, it is challenging to control parasites in animals in these areas due to the lack of financial resources by the population for the adequate treatment and control of fleas [3,25]. Because of this, people commonly treat animals with tungiasis using techniques that can sometimes harm their health, such as non-sterile removal [25,44]. As mentioned by Harvey et al. (2017), the instruments most used to remove fleas from dogs in an endemic area in Brazil included needles, pins, thorns, or pliers, used both for removing lesions in humans and animals [44]. This conduct predisposes to bacterial infections, increased inflammation, and in humans, potential transmission of viral pathogens such as HIV, hepatitis B, and hepatitis C [45,46]. In animals, complications such as concomitant infestation by other parasites (e.g., myiasis) can also occur in tungiasis lesions, acting as an aggravating factor [16], as observed in Figure 4. Thus, the challenges in controlling animal tungiasis also comprise the animal’s well-being, which is sometimes seriously affected [11]. Disease control in animals considered reservoirs, such as dogs, can be a viable option to control tungiasis in endemic areas [44]. However, there are few clinical studies evaluating ectoparasiticides’ effectiveness against *T. penetrans* to control and treat infections in domestic animals [35,36,37,38,39]. Thus, conducting new tests is vital to develop effective and economically viable options for society.

## 3. Tested Treatments for Animal and Human Tungiasis

The prominence of dogs and pigs in tungiasis has been evident since the first attempts at treating the disease. The first insecticides tested were based on organochlorines [26,27]. In 1967, lindane (gamma-hexachlorocyclohexane) was used topically on pigs infected with *T. penetrans* [26]. In 1976, another report of the use of organochlorines in infected pigs was also described, with resolution of the cases [27]. These works reported the elimination of fleas with these drugs, but this class of pesticides is currently prohibited in many countries due to its toxicity and environmental contamination [26,27]. These studies did not present data to support the prevention of new infections, and the authors only reported an improvement in the tungiasis condition of the animal host.

In 1989, a case report also described the use of trichlorphone 0.2% (Neguvon) in cases of infected dogs, with reduction in fleas in the animals studied [28]. More recently, in 2008, another study tested a 97% solution of trichlorphone in oil and found it ineffective against *T. penetrans* in dogs and cats. In the same study, collars impregnated with propoxur (carbamate) and flumethrin (pyrethroid) were also tested and showed low efficacy against *T. penetrans* in infected dogs [22].

After this period, some studies of pharmacological treatments against tungiasis were reported in humans, such as oral ivermectin. In 2003, a human study evaluated the topical use of ivermectin (0.8%), metrifonate lotion (trichlorfon, 0.2%), thiabendazole lotion (5%), and thiabendazole ointment (5%). The authors observed that these active principles could significantly reduce the number of lesions. The groups were evaluated 3, 7, and 12 days after treatment, and on day 12, the authors reported that almost all fleas were dead. A decrease in viable lesions was observed during this period, with no significant difference between the treatment groups. However, the authors pointed out that further studies would be needed to optimize the doses and administration of these medications [47]. Another study, in 2004, demonstrated that oral ivermectin for humans did not show significant clinical efficacy against *T. penetrans* at the administered dose (300 µg/kg of body weight in a single dose, repeated after 24 h) [48].

In dogs, ivermectin for treatment of tungiasis was used for some time in isolated infections, as in the case report of a dog with tungiasis, in which ivermectin (Ivomec^®^ 1% injection solution) was administered subcutaneously at a concentration of 0.3 mg/kg, causing total disappearance of the lesions about one month after treatment [31]. Ivermectin was also utilized in another case of infection in dogs in a rural area endemic to *T. penetrans*. In that study, the result after using the medication was not described [49].

Additionally, topical solutions tested in humans have proved to be effective in treating tungiasis, but their applicability and effectiveness have yet to be investigated in animals. They may also be economically viable options for the control of animal tungiasis [24,50,51,52], as demonstrated in a study of human tungiasis in 2009, with topical application of coconut oil (80%) associated with neem seed oil (20%) in the NC group and bathing the feet of patients with KMnO4 (single treatment; 15 min application on day 1). Both treatments led to the elimination of fleas, although neem and coconut oil contributed to significant clinical improvement in acute pathology. On average, 67% of all live fleas in the NC group and 37% in the KMnO4 group showed abnormal development (early senility). Additionally, 64% of the patients treated with KMnO4 and 78% of those treated with NC showed a reduction in pain. Regarding itching, 67% of patients treated with NC and 60% with KMnO4 reported a decrease in itching within seven days. However, the neem/coconut oil mixture used in that trial was no longer effective in killing embedded *T. penetrans* after seven days, killing an average of 30–40% of fleas within six days [52].

Another example was a study conducted in Madagascar in 2013, which evaluated the effectiveness of applying an active plant-based repellent (Zanzarin^®^) against *T. penetrans* in infected humans. The formulation proved to be effective, and the intensity of the infestation decreased during the 10-week observation period, achieving an attack rate of zero (median) after using the repellent. Furthermore, the morbidity associated with tungiasis was reduced to an insignificant level [24]. However, the product is not commercially available in tungiasis-endemic countries.

In 2017, the topical administration of NYDA, an association of two dimethicones, was also evaluated against human tungiasis. The results revealed that seven days after treatment, 78% of those treated lost all signs of flea viability, and 90% of penetrated fleas showed abnormal development five days after treatment. In general, there were decreased signs of inflammation in the group treated with NYDA [51]. Mutebi et al. (2021) cited the treatment of a goat with paws infected by *T. penetrans* with the same active principle and obtained a positive result, as shown by the images presented in that study. Parasite death was described two days after using the formulation [11]. However, the authors stated that studies of the effectiveness of the formulation were needed before it could be recommended for animal use. Sometimes, effective drugs against human tungiasis are used to treat animal tungiasis due to the lack of commercial formulations tested for the treatment and control of this disease in domestic animals.

## 4. Advances in Treatments of Animal Tungiasis

Concerning the treatment of animal tungiasis, a study conducted in 2005 evaluating the combination of 10% imidacloprid and 50% permethrin (Advantix^®^) demonstrated effectiveness, according to the researchers, of this formulation against tungiasis lesions in dogs. In the field trial, 17 dogs infected with *T. penetrans fleas* were topically treated with the Advantix^®^ formulation, while 17 remained untreated. Seven days after treatment, the authors observed a lower flea load in the treated dogs. An efficacy of 80% was achieved in the group treated on day 14 and 86% on day 21, but on day 28, there was already a decrease to 53%, while all dogs in the control group were parasitized. So, most dogs were free of tungiasis lesions in the treated group, while in the untreated group, the flea burden remained high [29].

Additionally, a case report described the treatment performed on a dog with lesions compatible with tungiasis identified on pads. The lesions were surgically removed, with the subsequent daily use of fipronil spray for seven days. There was complete recovery, but it was impossible to infer whether there was efficacy in the treatment with fipronil, since it was applied after the removal of the lesions [53].

In 2016, a topical aerosol containing chlorfenvinphos 4.8%, dichlorphos 0.75%, and gentian violet 0.145% (Supona^®^ aerosol) showed some tungicidal efficacy by improving the morbidity associated with tungiasis in pigs. The study evaluated two groups, 29 in the treatment group and 26 in the control group. One week after treatment, 58.6% of treated pigs had no viable lesions compatible with tungiasis, while all control pigs had at least one viable lesion. The study demonstrated that topical treatment was influential in the treatment of porcine tungiasis [30]. However, the study did not evaluate the residual period of the drug to infer how long the combination of active principles would be effective against *T. penetrans*. Evaluations were only performed on days 0 (pre-treatment) and 7 (post-treatment).

In 2016, an aerosol product containing 4.8% chlorfenvinphos, 0.75% dichlorphos and 0.145% gentian violet (Supona^®^ aerosol) was used to treat two severe cases of tungiasis in two infected goats in Uganda, Africa. In these cases, the kids presented viable lesions for tungiasis (stages II and III), and in the evaluation one week after the treatment, the animals no longer had viable lesions. They also had no clinical signs of bacterial infection and the hooves were in the process of re-epithelialization and moved normally. Despite reports of the tungicidal effect of Supona^®^ aerosol, this formulation was never validated through controlled studies for use in goats [54].

Regarding bovine tungiasis, a report of cattle infected by *T. penetrans* described the topical treatment of the hooves and teats with the direct application of a 4% trichlorfon solution in the footbath, where all the cattle recovered in approximately 20 days. However, the remaining fleas in the environment possibly contributed to re-infection [55].

Tungiasis directly affects the productivity of livestock. Difficulty walking to feed; lesions in the mammary glands that make it impossible for the calves to feed, in turn causing malnutrition and inability to develop; infertility of males that have severe lesions in the testicles; and pain promote this drop in productivity [56,57].

Currently, the options of ectoparasiticides for the treatment of livestock infested with *T. penetrans* are limited. Hence, there is an urgent need to investigate the prophylactic effects of alternative formulations for the management of tungiasis in these animals, avoiding the decrease in their production and elimination of drug residues in their final products, with the overall goal of reducing the economic impacts of tungiasis in endemic regions severely affected by the disease.

Wild animals, as well as pets and livestock, can be directly affected by *T. penetrans* infections. The clinical signs are usually related to the severity of the infections and can interfere with the execution of common activities by these animals, such as difficulty in walking, making these animals more susceptible to their predators [17,18,19,20], or hampering their ability to hunt for food [19].

Although studies on tungiasis in wild animals are limited, some reports have described treatments in different wild species rescued or referred for medical care at specialized centers. In 2017, an anteater (*Myrmecophaga tridactyla*) was rescued and multiple lesions on the paws caused by *T. penetrans* fleas were observed. The animal was treated with ivermectin at 0.2 mg/kg, subcutaneously (Ivomec^®^), with a second dose applied 14 days after the surgical removal of the lesions [17]. In 2022, a monkey (*Alouatta guariba clamitans*) infected with *T. penetrans* was treated with topical application of 5.7 mg of nitenpyram (Capstar^®^) in a single dose, together with ivermectin at a dose of 0.2 mg/kg subcutaneously and 2.5 mL (SC) of anti-tetanus serum [20]. Both treatments caused improvement of lesions, but the authors did not report how many days it took after treatment for the animals to be free of viable lesions.

Although some previous works have reported effectiveness with the mentioned concentrations, the drugs cannot be considered to have clinical relevance as antiparasitics since, according to the guidelines for evaluating the effectiveness of antiparasitic substances for the treatment and prevention of ticks and fleas in dogs and cats, the European Medicines Agency (EMA) defines that the efficacy of the standard product must be at least 95% for adult fleas and at least 90% for the inhibition of the emergence of adult fleas. In addition, they must be nontoxic to the treated animal, and in the case of tungiasis, they must be resistant to water (e.g., rain) [58].

## 5. A Promising Discovery for the Treatment of Animal Tungiasis

A recent study has revealed the efficacy of an active ingredient belonging to the isoxazoline group, fluralaner, which showed excellent systemic and persistent insecticidal efficacy. This active ingredient blocks γ-aminobutyric acid (GABA) and glutamate-dependent chloride channels in neurons, interrupting the conduction of inhibitory stimuli and causing exacerbated excitation, paralysis, and death of the parasites [34,59]. Fluralaner is a systemic acaricide and insecticide with prolonged efficacy in animals. The drug has commercial oral formulation in the form of palatable tablets at a dosage equivalent to 25–56 mg/kg of bodyweight for dogs [35].

In the study cited above, fluralaner’s therapeutic and residual efficacy was evaluated for treating dogs naturally infected with *T. penetrans* in a field trial challenge. Sixty-two dogs from an endemic community were randomized in a controlled double-blind study in which 31 dogs received oral fluralaner (*Bravecto*^®^ chewable tablets) at doses of 25 to 56 mg of fluralaner/kg and 31 animals (control group) received no treatment. After the administration of the formulation, more than 90% of the dogs in the treated group were free of *T. penetrans lesions* between days 14 and 90, and the treatment was 100% effective on days 21, 28, and 60. The efficacy was about 97% on day 90, dropping on day 120 to 84%, reaching 6% on day 150 against *T. penetrans*. The flea counts on *fluralaner-treated* dogs were significantly lower (*p* < 0.025) than on the control dogs at intervals from day 7 to 120. The authors of this study concluded that a single oral dose of fluralaner was effective for treatment, achieving long-term prevention (greater than 12 weeks) of tungiasis in dogs, without side effects on the animals [35].

In addition to evaluating the effectiveness of the Bravecto^®^ in a chewable tablet formulation, the study clinically evaluated all dogs using a canine acute tungiasis severity score (SCADT), which related clinical signs common to tungiasis, such as pain, hyperemia, erythema, among others, as well as the number of viable lesions caused by *T. penetrans* according to the experimental group. It was observed that from day 7 to day 120, the mean SCADT scores were significantly reduced in treated dogs, with an average of 0.10 compared to 1.54 at day 120 for untreated dogs [35].

## 6. Conclusions

Since human and animal tungiasis are closely related, the effective treatment of domestic animals for *T. penetrans*, especially in endemic areas, can improve control of the disease in humans. Despite the existence of many studies on the treatment of animal tungiasis, most of these studies have shortcomings, such as the failure to apply randomized blinded designs to avoid biases, preventing readers from defining, for example, the percentage of effectiveness of the formulations tested.

Given the therapies tested so far to combat tungiasis in dogs, one of the main reservoirs and dispersers of the flea in the environment, the results of efficacy have not demonstrated effectiveness of the use of most of the substances tested so far. However, research carried out with fluralaner has shown it to be an effective alternative, although its high cost in some places often makes its use unfeasible for some people living in endemic areas. Thus, new studies with other isoxazolines and active ingredients focused on evaluating the effectiveness against animal tungiasis should be carried out, since empirical studies describing treatments do not contribute to controlling this zoonosis. In addition to this class, phenylpyrazoles can also be an option for treating *T. penetrans*, such as the commercial product fipronil, which has a topical pulicidal effect. However, its effectiveness against *T. penetrans* has yet to be *evaluated*.

Additionally, it is important to notice that fluralaner and other commercial medications have been shown to be effective for use in companion animals; however, there are currently no commercial products available that are effective for use in production for animals.

## Data Availability

Not applicable.
